# Improved Functioning and Activity According to the International Classification of Functioning and Disability after Multidisciplinary Telerehabilitation for Post-COVID-19 Condition—A Randomized Control Study

**DOI:** 10.3390/jcm13040970

**Published:** 2024-02-08

**Authors:** Indre Bileviciute-Ljungar, Jan-Rickard Norrefalk, Kristian Borg

**Affiliations:** 1 Department of Clinical Sciences, Karolinska Institute, Danderyd University Hospital, 18288 Stockholm, Sweden; norrefalk@hotmail.com (J.-R.N.); kristian.borg@ki.se (K.B.); 2 Multidisciplinary Pain Clinic, Capio St. Göran Hospital, 11219 Stockholm, Sweden; 3 Department of Physical and Rehabilitation Medicine, Danderyd University Hospital, 18288 Stockholm, Sweden

**Keywords:** post-COVID-19 condition, International Classification of Functioning and Disability (ICF), telerehabilitation, multidisciplinary

## Abstract

This study investigates the outcomes and feasibility concerning the functioning and activity of multidisciplinary group telerehabilitation for a post-COVID-19 condition. Recruitment for the group rehabilitation was announced three times during 2021 and 2022 through the COVID-19 patient organization in Sweden. The key inclusion criteria were remaining symptoms and functional impairments beyond 12 weeks after SARS-CoV-2 infection; medical assessment and treatment regarding comorbidities or new postinfection symptoms; the ability to use the Internet. Participants were randomized into a rehabilitation group or onto a waiting list using an Internet program. Multiple outcomes included self-scored questionnaires and physical tests before and after eight weeks, and at six months follow-up. Here, we present the self-scored outcomes on the International Classification of Functioning and Disability questionnaire (ICF, 22 body functions and 16 activity/participation categories) and breathing scales. Of the 164 participants who registered for the study, 67 (mean age 43, 78% women) participated in an eight-week group rehabilitation compared to 42 who served as waiting list controls (mean age 47, 88% women). At six months follow-up, 60 participants from the rehabilitation group and 21 from the waiting list completed the data. The results indicate that a larger number of ICF body functions and activity/participation categories had improved in the rehabilitation group after eight weeks and six months. Overall credibility, as assessed by the Credibility Expectancy Questionnaire, was high, and the attrition rate in rehabilitation was low. The results indicate beneficial outcomes for multidisciplinary telerehabilitation in people suffering from a post-COVID-19 condition. Therefore, rehabilitation interventions should be further developed and implemented in clinical practice.

## 1. Introduction

According to the World Health Organization (WHO), the number of people suffering from a post-COVID-19 condition might be as high as 10% of all infected cases [1]. In Sweden, the first epidemiological data collected until 15 February 2022 were based on ICD-10 diagnosis codes related to acute SARS-CoV-2 infection and a post-COVID-19 condition. These showed a prevalence of approximately 2% in a population of 4.1 million inhabitants [2]. In practice, the prevalence of a post-COVID-19 condition is expected to be even higher, since many inhabitants were undiagnosed with an acute SARS-CoV-2 infection during the first months in 2020. Moreover, the definition of post-COVID-19 condition was established in September 2021 [1].

Lingering ailments after a SARS-CoV-2 infection may include a variety of disabling symptoms related to the nervous system (fatigue, cognition, depression/anxiety, sleep, and pain) and the somatic organs (breathing, cardiovascular, abdominal, and muscle problems) [1]. This indicates a need for a rehabilitation approach that covers the entire range of symptomatology and disability. The first rehabilitation study for post-COVID-19 syndrome was performed as a face-to-face intervention for hospitalized patients, and it showed positive effects on fatigue and exercise capacity [3]. Most post-COVID-19 rehabilitation studies have focused on physical training [3,4,5,6], pulmonary rehabilitation [7], or a combination of these [8,9,10]. Some studies tested a multidisciplinary approach involving psychoeducation, psychotherapy, and physical training [10,11]. Health-related quality of life, and muscular, cardiovascular, and pulmonary parameters were chosen as the primary outcomes [3,8,11,12]. During ongoing pandemics, the rehabilitation clinics started to use Internet-based interventions, such as applications in smartphones [13], prerecorded sessions [6], and individual sessions with videoconferencing [12]. Some studies also included education in combination with other interventions [3,6]. Although relatively few, these rehabilitation studies reported improvements in muscular parameters measured by physical tests within the group [3,6,11] or compared to the control group(s) [5,9]. The cardiorespiratory outcomes were also improved compared to control group(s) [5,8,9]. However, to our knowledge, there is no report on multidisciplinary group telerehabilitation in a post-COVID-19 condition.

One of the ways to acquire a report of a person’s functioning, activity, and participation is to use an International Classification of Functioning, Disability, and Health (ICF), approved by the WHO in 2001 [14]. The ICF is a valuable instrument for gathering data that covers multiple symptoms and allows one to evaluate how they affect the body’s functioning and activities/participation. However, reports on using the ICF in a post-COVID-19 condition are rare and more studies are needed [15,16].

The aim of the present study was to investigate the short- and long-term outcomes, appropriateness, and feasibility of the multidisciplinary telerehabilitation conducted in groups for people with symptoms that remain after a SARS-CoV-2 infection (post-COVID-19 condition). Our hypotheses were as follows: (1) multidisciplinary telerehabilitation in a group is a suitable and effective way to improve the functions and activities in people suffering from a post-COVID-19 condition; (2) the positive effects of telerehabilitation will still be present at six months follow-up. Primary outcomes were the functioning and activity according to the ICF questionnaire. Evaluation of breathing scales were chosen as secondary outcomes in order to understand the results in regard to other rehabilitation studies, which mainly focused on lung functions in post-COVID-19 patients [8,17].

## 2. Materials and Methods

### 2.1. Participants

Participants were recruited via Facebook sites and stakeholder organizations for post-COVID-19 syndrome in Sweden (i.e., Svenska Covidföreningen). An online announcement was published alongside a further link for detailed information. All participants who signed an agreement, filled in questionnaires, and performed physical tests with a pulsoximeter, were included in the randomization, as performed by an Internet program at RANDOM.ORG., accessed on 5 May 2021, 27 August 2021 and 28 April 2022. Thereafter, the participants were informed by an email regarding the results of the randomization and were asked again to confirm their participation in the study. The waiting list controls were informed that they would be offered telerehabilitation in the next round. Inclusion criteria were as follows: (a) COVID-19 infection supported by medical history and/or positive tests for the COVID-19 virus (PCR) and/or a positive immunoglobulin response; (b) age between 18 and 70; (c) significantly reduced level (at least 50%) of functioning and activity/participation in daily life after infection; (d) persistent symptom duration at least 12 weeks after acute infection; (e) medically assessed participants with satisfactorily managed comorbidities or new postinfection symptoms; (f) the ability to use the Internet, complete Internet-based questionnaires, and participate in a rehabilitation program delivered through “Teams” (Microsoft program) for a group comprising a maximum of 25 participants over an 8-week period. Exclusion criteria were as follows: (a) unclear onset of symptoms in relation to COVID-19 (for example, stress factors, post-traumatic stress disorder, other types of psychological and somatic trauma in combination with a COVID-19 infection or before it); (b) abuse of alcohol or psychotropic substances; (c) diagnoses of psychological or somatic conditions that are unstable and require appropriate treatment (e.g., hypothyroidism, lung, heart, kidney diseases, psychosis, suicidality, etc.); (d) ongoing psychological and medical treatments that may interfere with the rehabilitation (for example, other psychotherapies or adjustment of pharmacological drugs).

All participants completed questionnaires at the start and after eight weeks through the online platform BASS at the eHealth Core Facility at Karolinska Institutet. The rehabilitation group completed questionnaires even at six months follow-up. The ongoing pandemic resulted in difficulties starting rehabilitation for waiting list participants after six months, which was initially planned to create follow-up data. Later, an adjustment to the ethical permissions was obtained to collect data for those controls who did not undergo rehabilitation after being on the waiting list. Therefore, we were able to collect less six-month follow-up data than planned.

### 2.2. Questionnaires

To measure the outcomes reflecting a variety of postinfectious symptom manifestations, we chose ICF categories and breathing scales.

The Functional Compass COVID-19 questionnaire is a self-assessment questionnaire developed from the Functional Barometer [18] adapted to the ICF regarding patients’ disability, activity, limitations, and participation [19]. The Functional Compass COVID-19 questionnaire consists of 47 questions, and the questions are formulated to self-assess functions regarding impairments in body functions, such as respiratory, cardiovascular, thermoregulatory, olfactory, gustatory, tactile perceptions, muscular, and joint functions. All items were assessed by a verbal descriptive problem scale, the same as the ICF qualifier; all five categories were graded between 0 and 4. The categories are defined as no (0), light (1), moderate (2), severe (3), and total (4) problems [19].

In the present study, we analyzed and presented 38 categories based on the ICF (22 b-categories and 16 d-categories); for ICF codes, please see Supplementary Material S1. We excluded 4 categories based on the same ICF category b280, “Sensation of pain”, and 4 categories related to skin sensation, as well as the optional item variable “What would you like to do if you felt a little better?”. The Functional Compass COVID-19 questionnaire has not been validated among post-COVID-19 conditions and was collected as self-scored data.

The modified Medical Research Council Dyspnea Scale (mMRC) is a self-rating tool to measure the degree of disability that breathlessness poses during day-to-day activities on a scale from 0 to 4 (https://www.mdcalc.com/calc/4006/mmrc-modified-medical-research-council-dyspnea-scale, accessed on 15 January 2021). This scale is broadly used to measure dyspnea in research and clinics, but has a so-called ceiling effect [20]. Recently, Zhang and colleagues reported a moderate correlation between the Brief ICF core set for Chronic Obstructive Pulmonary Disease (ICF-COPD) and the mMRC [21]. Two of four b-categories and three of four d-categories of the Brief ICF-COPD are also included in the Functional Compass COVID-19 questionnaire.

The Clinical COPD questionnaire (CCQ https://ccq.nl, accessed on 15 January 2021) has been developed to measure the clinical disease control of patients with Chronic Obstructive Pulmonary Disease. It has 10 items, each using a 7-point scale from 0 (asymptomatic) to 6 (extremely symptomatic/totally limited). The total score of the domains is calculated by adding all the scores together and dividing this sum by the number of questions. The CCQ was validated in a Swedish cohort with good validity [22].

The Credibility Expectancy Questionnaire (CEQ) (https://www.clintools.com/victims/resources/assessment/rct/ceq.pdf, accessed on 15 January 2021) consists of three credibility and three expectancy items regarding the treatment. Total scores range from 0 (not at all credible/no expectancy for improvement) to 100 (very credible/large expectancy for improvement). The CEQ has good psychometric properties with a standardized alpha of r = 0.85 (for both scales) [19]. Participants completed the CEQ in the middle and at the end of rehabilitation. However, no report regarding validation in a Swedish cohort has been found.

The negative effects were captured during 5–10 min of feedback at the start of each session and during individual weekly appointments with team members as well as at the end of the rehabilitation study.

### 2.3. Telerehabilitation Programme

No guidelines were available for a post-COVID-19 condition rehabilitation at the beginning of 2021. A digital approach (telerehabilitation) for participants with remaining post-COVID-19 symptoms was chosen due to pandemics, social restrictions, and the clinical experience of the team members, adapting the content from the previous telerehabilitation programs used in the clinical setting at the Multidisciplinary Pain Clinics, Capio St. Göran hospital, Sweden.

The first aim of the interventions was to normalize the autonomic nervous system activity by using breathing exercises, mindfulness, and an ACT approach. The second aim was to normalize body functions by regular (tailored) exercises during the program and using the ExorLive Go application (https://www.exorlive.com/uk, accessed from 1 May 2021). The third aim was to create an individual rehabilitation plan for each participant in order to individualize the rehabilitation process. The physical part of the program included relaxation and muscle strength training, such as yoga and Qigong. Exercises using compassion and DoIn approaches were other interventions that strengthened the mental self-compassion of the participants. Psychoeducation regarding symptoms’ pathophysiology and management was performed by multidisciplinary team members.

A multidisciplinary 8-week telerehabilitation program in the group was performed by team members using the “Microsoft Teams” platform (https://www.microsoft.com/en-us/microsoft-teams/group-chat-software, version “classic”, accessed from 1 May 2021). This included weekly six-hour telerehabilitation sessions (three days/week, two hours per session). Additionally, three hours of exercise on one’s own using videorecorded exercises in the ExorLive Go application, or other free-chosen physical activities (walking, jogging, etc.), was encouraged to be registered every week. The physiotherapist supervised the ExorLive Go application during the eight-week telerehabilitation program. Six individual sessions, meeting every team member at least once, were also offered with the aim to formulate individual rehabilitation goals as a part of the rehabilitation process. The presentation of group interventions given through the “Microsoft Teams” platform are summarized in Supplementary Material S2. After the program, the participants continued to have access to the ExorLive Go application, but were not followed by the physiotherapist. The study was registered on ClinicalTrials.gov, identifier: NCT04961333, and approved by the Swedish Ethical Authorities (Etikprövningsmyndigheten), Dnr. 2020-07216.

### 2.4. Statistics

Descriptive statistics for nominal data (genus, origin, education, sick leave, etc.) are presented as the number of participants and percentage per group. Age and body mass index (BMI) are presented in mean, standard deviation, and range. Descriptive statistics was used for the ICF qualifiers, ranging from 0 to 4 [14].

For qualitative data (sex, origin, education, sick leave, etc.), the chi-square test was used to compare any significance variations between the rehabilitation group and waiting list. For normally distributed parameters (age and BMI), an independent *t*-test was used. For ordinal parameters (ICF categories, mMRC, and CCQ), a nonparametric Mann–Whitney test was used to assess the statistical significance between the groups. A Wilcoxon test was used to analyze data for within-group differences between two time measurements, and a nonparametric Friedman test was employed for three time measurements. A general linear model was used to identify the role of age and group in the ICF categories measured over time. Time was chosen as a within-subjects factor, group as a between-subjects factor, and age as a continuous covariate. The statistical package SPSS, version 27, was used for analyses.

## 3. Results

### 3.1. Sociodemographic Data

**Figure 1:**
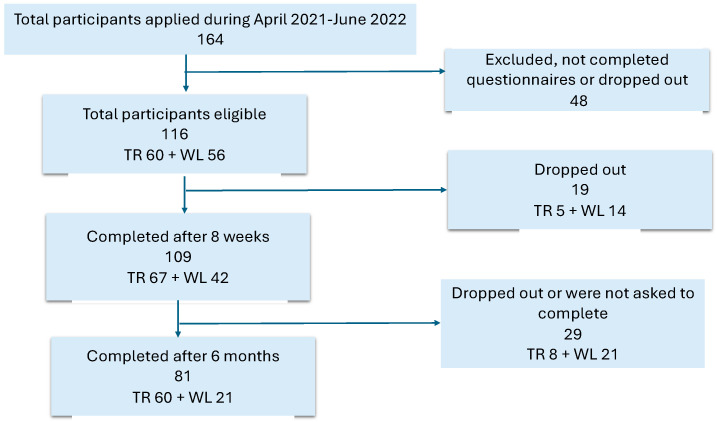
Flow chart of the study cohort. No differences have been found between the groups, except for age in the final cohorts (independent sample test, see Supplementary Material S3). Abbreviations: TR = telerehabilitation group; WL = waiting list.

The recruitment and randomization flow-chart are presented in Figure 1. Briefly, 164 participants registered for the study. Those who completed the questionnaires and performed physical tests to exclude desaturation (lower than 90% during the tests) were eligible for the randomization. After the randomization, 116 participants continued to participate, and 109 participants completed questionnaires after eight weeks, as shown in Figure 1. More information regarding age, sex, and BMI distributions is presented in Supplementary Material S3. No difference in age, sex, and BMI have been identified between the separate recruitments.

The baseline characteristics of the final cohort of 109 participants are presented in Table 1. A large number of participants were middle-aged (mean 45 years) females (82% women). More than half of the sample had a college or university degree, and about half were living in a registered partnership/marriage. Most participants were employed, and more than half of the participants were working during the study. Approximately 50% of participants were on sick leave, disability pension, or received social security payments. Only one participant smoked, and none used narcotic substances during the study period. Regarding alcohol intake, a weekly amount calculated in wine glasses was less than one for the telerehabilitation group and less than two for those on the waiting list.

When comparing the telerehabilitation and waiting list control groups, the only difference found between the groups was a higher age in the final waiting list cohorts compared to the rehabilitation groups after eight weeks and six months.

### 3.2. Clinical Data

The symptom duration was calculated in accordance with the participants’ reported data on the start of symptoms or the date of the PCR test. Results showed a 51-week (SD 27) symptom duration for the telerehabilitation group, with a 58 (SD 31)-week duration for those on the waiting list, and no significant difference between the groups (independent t-test, results not shown).

A SARS-CoV-2 PCR test was taken by 59% of participants in the telerehabilitation group and 41% of those on the waiting list group, with no difference between them (chi-square test, *p* = 0.18). Among those who performed the test, 63% tested positive in the telerehabilitation group compared to 38% in the waiting list group (chi-square test, *p* = 0.3). An antibody test (AT) was performed by 60% in the telerehabilitation group and 40% in the waiting list group (chi-square test, *p* = 0.4). Among those who performed an AT test, 52% were positive in the telerehabilitation group compared to 48% in the waiting list (chi-square test, *p* = 0.19). Only seven (10%) participants in the telerehabilitation group and six (14%) on the waiting list were hospitalized during acute SARS-CoV-2 (chi-square test, *p* = 0.4).

Approximately 30% of participants reported having a chronic disease before a SARS-CoV-2 infection; see Table 2. Asthma and psychiatric diseases were most common before infection and varied between 8 and 10%; see Table 2. After the infection, and when applying for the study, there was a notable increase in the medication regarding cardiovascular, antiasthmatic, psychiatric, anti-inflammatory, sleep-regulating drugs, and pain killers or pain-modulating drugs when referring to information concerning the chronic disease prior to the infection; see Table 2. Almost 25% of participants in both groups reported taking medication for asthma, and over 30% reported cardiovascular system-affecting drugs, many of which were beta-blockers. Prescribed psychiatric drugs modulating descending pain pathways (i.e., duloxetine, amitriptyline, and mirtazapine) were also taken by more than one-quarter of the participants; see Table 2. Increased intake of prescribed sleep medication, gastrointestinal drugs, and vitamins were also reported, although few reported corresponding diseases present before a SARS-CoV-2 infection; see Table 1. No significant differences were obtained between the groups regarding comorbidities before the SARS-CoV-2 infection and medication at the start of the study.

### 3.3. Functional Compass COVID-19 Questionnaire after Eight Weeks

**Figure 2:**
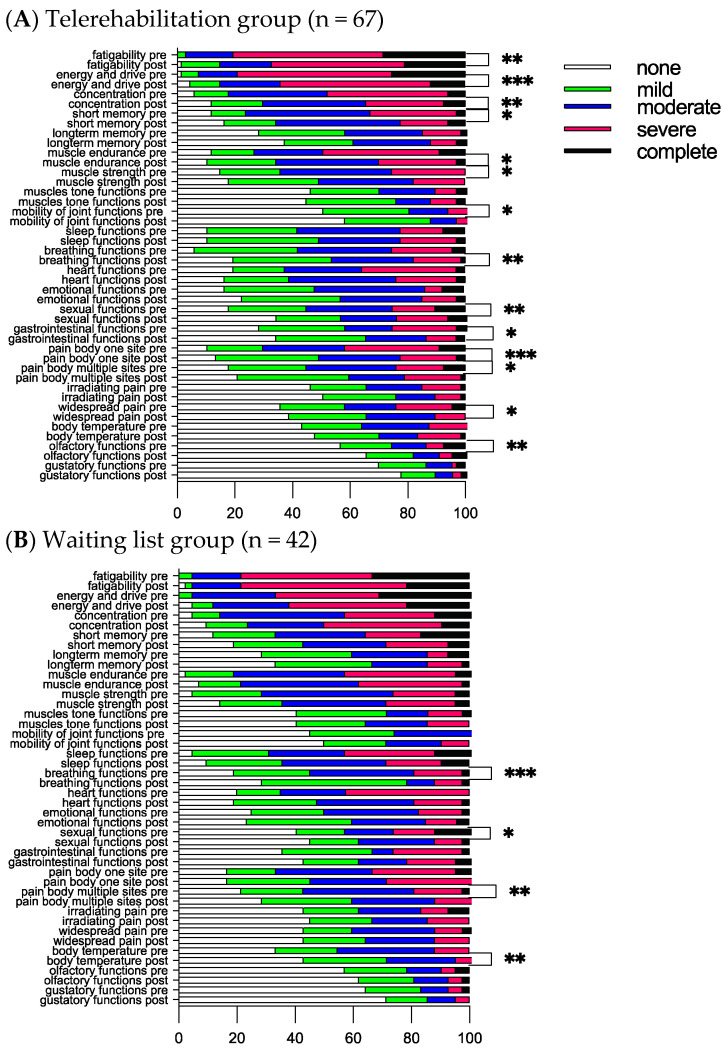
Twenty-two ICF b-categories are presented as frequencies at the start (pre-) and after 8 weeks (post-). * Indicates significant differences within the group using the nonparametric Wilcoxon signed rank test; * *p* < 0.05, ** *p* < 0.001, and *** *p* < 0.001.

The most impaired functions in the study cohort were neurocognitive/mental functions (fatigability, energy and drive, concentration, short/long-term memory, and sleep), muscular (muscular endurance and muscle strength), breathing, heart functions, and pain-related categories (pain in body at one site and pain in body at multiple sites); see Figure 2. No significant differences were obtained between the groups at the start, except for a tendency to experience more impaired sleep among those on the waiting list (*p* = 0.056, Mann–Whitney U test). In both groups, breathing functions were found to be significantly improved from a median score of two to one after eight weeks. Participants on the waiting list scored more improvements in breathing functions as compared to those in the telerehabilitation group (Mann–Whitney test, *p* = 0.034). Apart from breathing functions, the following improvements were obtained within the groups for both groups: sexual functions and pain body at multiple sites (Figure 2). For the telerehabilitation group, additional improvements were obtained in cognitive/mental functions, such as fatigability, energy and drive, concentration, short memory, muscle endurance and strength, mobility of joint functions, gastrointestinal functions, pain in the body at one site, widespread pain, and smell (Figure 2A). For the waiting list, an additional improvement was obtained in body temperature (Figure 2B), while in the telerehabilitation group, there was a strong tendency for improvement (*p* = 0.051).

**Figure 3:**
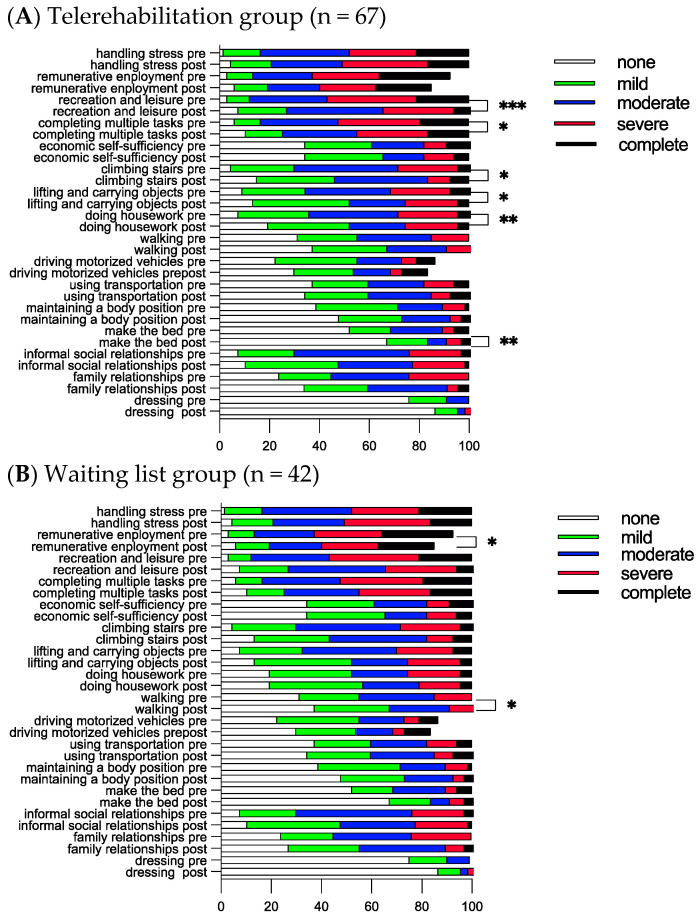
Sixteen ICF d-categories are presented as frequencies at the start (pre-) and after 8 weeks (post-). * Indicates significant differences within the group using the nonparametric Wilcoxon signed rank test; * *p* < 0.05, ** *p* < 0.001, and *** *p* < 0.001.

For the ICF d-categories, the most impaired activities among the participants in the study cohort were social-, mental-, work-, and household-related activities (completing multiple tasks, handling stress and other psychological demands, informal social relationships, remunerative employment, and doing housework) and physical activities (climbing stairs, and lifting and carrying objects) (Figure 3). No significant differences were obtained between the groups at the start and after eight weeks. Participants in the telerehabilitation group scored improvements in recreation and leisure, completing multiple tasks, climbing stairs, lifting and carrying objects, doing housework, and making the bed (Figure 3A). The waiting list scored significant improvements in remunerative employment and walking (Figure 3B).

### 3.4. Functional Compass COVID-19 Questionnaire at Six Months Follow-Up

**Figure 4:**
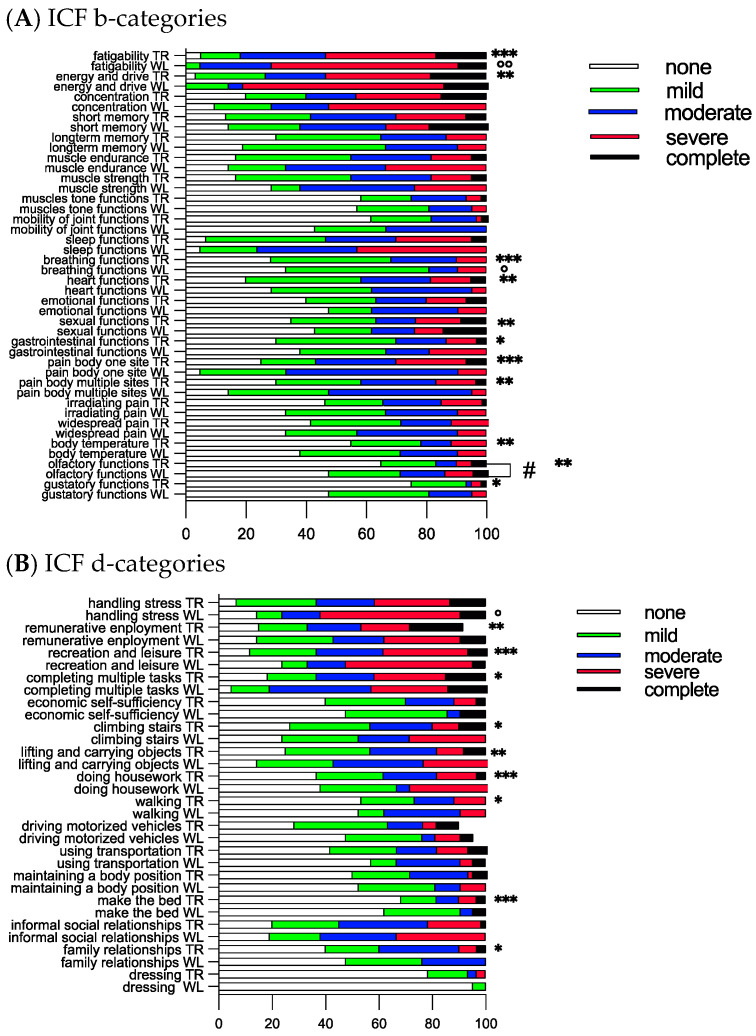
Twenty-two ICF b-categories and sixteen d-categories are presented as frequencies in telerehabilitation (TR) and on the waiting list (WL) at 6 months follow-up. * Indicates significant differences within the telerehabilitation group (n = 60), ° within the waiting list (n = 21), and # between the groups. A nonparametric Friedman test within the groups and Mann–Whitney test between the groups were used. * *p* < 0.05, ** *p* < 0.001, and *** *p* < 0.001; ° *p* < 0.05; °° *p* < 0.01 and # *p* < 0.05.

At six months follow-up, body functions (ICF b-categories) were still impaired for a large portion of cohort participants (Figure 4A). No significant differences were found between the groups, except significantly impaired olfactory functions (b1562) among those on the waiting list compared to those in the telerehabilitation group (Mann–Whitney test, *p* = 0.021) (Figure 4A). At the start and after eight weeks, the olfactory functions were scored as not impaired (median 0, range 0–4) both for the waiting list and telerehabilitation group. After six months, these functions were scored as mild impaired (median 1, range 0–4) by those on the waiting list, while the telerehabilitation group scored them as not impaired (median 0, range 0–4). Significant improvements in fatigability, energy and drive, breathing, cardiovascular, sexual, gastrointestinal, body temperature, and pain functions were obtained in the telerehabilitation group (Figure 4A). The waiting list scored improvements in fatigability and breathing functions (Figure 4A).

For activities and participation, there was no significant difference between the groups. The telerehabilitation group scored significant improvements in remunerative employment, recreation and leisure, completing multiple tasks, climbing stairs, lifting and carrying objects, doing housework, walking, making the bed, and family relationships (Figure 4B). The waiting list scored significant improvements in handling stress and other psychological demands (Figure 4B), while the telerehabilitation group had a tendency for improvement in this category (Mann–Whitney test, *p* = 0.072).

### 3.5. Regression Analysis

The ICF categories impaired in at least of 75% of participants that showed significant improvements within the groups during the study period were analyzed with a general linear model for repeated measurements. The results presented in Table 3 show the remaining severe impairments in energy (fatigue and fatigability); moderate impairments in cognitive functions and activities (concentration, short memory, handling stress, and completing multiple tasks), muscular endurance, and pain at one site after six months. Moderate restrictions were also found in work, social and leisure activities after six months. Several categories were improved and scored as mild impaired at six months follow-up: muscular strength, heart and breathing functions, pain in multiple sites, climbing stairs, lifting and carrying objects, and doing household. Regression analysis shows that time was a major factor for the improvements in muscle endurance, pain, and climbing stairs at eight weeks. Age influenced improvements in heart functions at eight weeks and six months follow-ups, as well as remunerative employment at eight weeks follow-up (Table 3).

### 3.6. Questionnaires mMRC and CCQ

Data for breathing scales are presented in Table 4, indicating mild-moderate breathing symptoms. No differences have been observed between the groups at the start, after eight weeks, or at six months follow-up. Briefing scales (mMRC and CCQ) showed a significant improvement after eight weeks in both groups, except for CCQ on the waiting list (Table 4). At six months follow-up, the breathing symptoms significantly improved from mild-moderate to mild in both groups (Table 4).

### 3.7. Adherence, Attrition, Credibility, and Negative Effects in the Rehabilitation Groups

The overall adherence to rehabilitation interventions was high for the whole study population: 92% (SD 11) participation in the sessions was registered by staff, with no differences between the three separate rounds during 2021 and 2022. Almost 75% of participants participated on 21–24 occasions, with the remaining 25% participating on 17 and 20 occasions.

During the first and the second rehabilitation rounds (2021), the participants were offered 24 sessions of group rehabilitation, and during the third round (2022), 22 sessions. The smaller number of maximum rehabilitation occasions during 2022 was related to one additional holiday in Sweden compared to the spring of 2021, and another one which was not compensated compared to the same period 2021. Therefore, there was no difference in participation between the first and the second rounds (median 23 occasions for both groups) (2021), while fewer occasions were registered for the third round (median 21) (2022) compared to the first and the second rounds (Mann–Whitney test, *p* < 0.01 and *p* < 0.001, respectively).

Analysis of the ExorLive Go application data showed that 64 participants (96%) used the application and reported individual physical activities with an average number of 4.1 (SD 2.5) hours of registered physical activity per week and participant. This was in line with a recommendation to record up to three hours of individual physical activity per week during the study period.

Attrition from the study was low within the telerehabilitation groups. Since randomization was performed after completing questionnaires, all participants provided initial data. Four participants (7%) from the telerehabilitation group withdrew. Additionally, one participant was registered as participating only five times and was excluded from the study. Those who participated in an eight-week telerehabilitation provided post-treatment data after eight weeks and 88% at six months follow-up. Among those seven participants who did not provide data at six months follow-up, three did so for medical reasons (new disorders) and four dropped-out by not responding to email communication or reminders. For individual appointments with team members, the median was five appointments out of six offered. A larger number of participants (70%) participated in five or six appointments.

The results of the Credibility and Expectancy (CEQ) scale were measured at four and eight weeks during the rehabilitation period, and are presented in Supplementary Material S4. Credibility and expectancy were high at both measurements and increased after eight weeks. No persistent negative effects have been reported after the study. During telerehabilitation, some participants did report a worsening of symptoms and were encouraged to perform the activities at a lower intensity during the next session. No one withdrew from the study due to temporary worsening of the symptoms.

## 4. Discussion

This is the first study on multidisciplinary telerehabilitation for a post-COVID-19 condition. Results indicate that the delivery of multidisciplinary telerehabilitation is a safe intervention and can be used for group telerehabilitation. During an ongoing pandemic, it might be the only safe and energy-sparing way to engage participants for a longer intervention period. The results of the ICF categories in the present study show more improvements in the telerehabilitation group than in the waiting list controls, particularly regarding different activities and participation. However, no differences have been found between the groups, except for more improved breathing functions after eight weeks and more impaired olfactory functions among those on the waiting list after six months. Despite a greater improvement in the ICF breathing categories after eight weeks, the waiting list did not score the corresponding improvements in breathing scales. This might be explained by the fact that participants in the telerehabilitation group became more aware of their breathing difficulties, since interventions related to breathing were one of the major focuses during telerehabilitation. Difficulties regarding impaired breathing functions were also discussed during telerehabilitation. The difference in breathing functions between the groups disappeared at six months follow-up. In contrast, the waiting list controls scored more impaired olfactory functions after six months, indicating some progression of neurological symptoms.

However, the results of this study clearly indicate that a post-COVID-19 condition is quite stable over the time regarding self-scored functioning and disability, and characterized by fatigue, fatigability, sleep disorders, and neurological symptoms even six months after telerehabilitation interventions or waiting for them. This suggests the chronicity of a post-COVID-19 condition in many participants, despite improvements in functioning and activities and the breathing scales. The results are in line with a recent prospective study of 548 participants reporting only 7.6% recovery almost 2 years after the infection [23]. Growing evidence indicate similarities between post-COVID-19 condition and myalgic encephalomyelitis/chronic fatigue syndrome (ME/CFS) [24,25]. Reports suggest applying knowledge within ME/CFS for post-COVID-19 condition [26]. At the same time, treatment studies for post-COVID-19 might be tested in patients with ME/CFS, including multidisciplinary group telerehabilitation, as reported in this study.

The present cohort was dominated by highly educated middle-aged women after mild infection. No initial differences in sociodemographic parameters and ICF categories have been found initially between the groups. However, after eight weeks and six months follow-up, the waiting list consisted of participants with a higher age as compared to the telerehabilitation group. Regression analysis showed that age influenced improvements in heart functions and remunerative employment. The higher age on the waiting list at follow-up might be explained by the fact that younger participants were not interested in being on the waiting list, possibly searching instead for outside help. During spring 2021, the Swedish National Board of Health and Welfare granted rehabilitation in primary health care for nonhospitalized sufferers of a post-COVID-19 condition [27]. Therefore, the results of the six months follow-up should be interpreted with caution, since participants might have received other interventions in primary health care after the eight-week telerehabilitation or waiting list period.

The strengths of the study were: (1) the randomization; (2) three separate rounds of recruitment and rehabilitation; (3) a waiting list; (4) individual appointments with a tailored approach for each participant and individual rehabilitation goals during the study. Moreover, the study was conducted in a clinical setting, which confirms the suitability for clinical adaptation. The online approach also allowed participants living in different parts of Sweden to participate, which was highly appreciated.

Limitations of the study: (1) only participants able to use the Internet and speak fluent Swedish were included and (2) the results should be compared with in-person rehabilitation. In this study, we present only self-scored data. The idea of an online approach for the study can be seen as a limitation, since no access to controlled medical recordings was available.

The next step in the study is to analyze the return-to-work process of the participants one year after the completed eight-week telerehabilitation and compare this to the waiting list or no interventions.

## 5. Conclusions

In conclusion, the results show that multidisciplinary telerehabilitation for a post-COVID-19 condition results in beneficial outcomes in functioning and activities/participation. However, the multidisciplinary telerehabilitation in people suffering from a post-COVID-19 condition needs further development to achieve greater improvements, as many participants still report disabling symptoms at six-months follow-up.

## Figures and Tables

**Table 1:** Detailed sociodemographic data at the start of study. * indicates difference between telerehabilitation group and waiting list; *p* < 0.05 and *** *p* < 0.001.

Parameters	8-Week Participants		6-Month Participants	
	Telerehab (n = 67)	Waiting List (n = 42)	Telerehab (n = 60)	Waiting List (n = 21)
Age (years), mean (SD)	43 (9)	47 (9) *	43 (8.6)	50 (7) ***
BMI (kg/m^2^), mean (SD)	25 (4.3)	27 (5.6)	26 (5.5)	26 (3.9)
Sex:				
Female	52 (78%)	37 (88%)	49 (82%)	18 (86%)
Male	15 (22%)	5 (12%)	11 (18%)	3 (14%)
Place of birth:				
Sweden	62 (93%)	37 (88%)	57 (95%)	17 (81%)
Outside Sweden	5 (7%)	5 (12%)	3 (5%)	4 (19%)
Marital status:				
Married	32 (48%)	20 (48%)	30 (50%)	12 (57%)
With partner	21 (31%)	10 (24%)	19 (32%)	3 (14%)
Single	14 (21%)	12 (28%)	11 (18%)	6 (29%)
Children:				
Yes	47 (70%)	32 (76%)	43 (72%)	18 (86%)
No	30 (30%)	10 (24%)	17 (28%)	3 (14%)
Living circumstances:				
Condominium	9 (13%)	7 (17%)	8 (13%)	5 (24%)
Own house	33 (49%)	26 (62%)	31 (52%)	13 (62%)
Rental house	21 (31%)	5 (12%)	17 (28%)	2 (9%)
Inherent	1 (2%)	0	1 (2%)	0
Other	3 (5%)	4 (9%)	3 (5%)	1 (5%)
Education:				
Primary (<9 years)	0	0	0	0
Secondary (10–12 years)	17 (25%)	9 (21%)	15 (25%)	3 (14%)
Higher (>12 years)	46 (69%)	28 (67%)	41 (68%)	15 (72%)
Other education	4 (6%)	5 (12%)	4 (7%)	3 (14%)
Employment situation:				
Working right now	37 (55%)	26 (62%)	33 (55%)	15 (71%)
Employed	53 (79%)	38 (91%)	50 (83%)	19 (91%)
Jobseekers	7 (10%)	2 (5%)	5 (8%)	2 (10%)
Studying	4 (6%)	1 (2%)	2 (3%)	0
No gainful employment	3 (5%)	1 (2%)	3 (5%)	0
Financial security:				
Sick leave, 25%	1 (1%)	0	1 (2%)	0
Sick leave, 50%	7 (10%)	1 (2%)	7 (12%)	0
Sick leave, 75%	1 (1%)	1 (2%)	1 (2%)	0
Sick leave, 100%	16 (24%)	13 (31%)	15 (25%)	6 (29%)
Disability pension, 50%	3 (5%)	0	3 (5%)	0
Disability pension, 100%	7 (10%)	1 (2%)	6 (10%)	1 (5%)
Unemployment benefits	5 (7%)	0	3 (5%)	0
Student aid	1 (1%)	0	0	0
Social security	1 (1%) *	4 (9%)	1 (3%)	2 (10%)
Other	1 (1%)	2 (5%)	1 (3%)	1 (5%)

Abbreviation: Telerehab = Telerehabilitation; BMI = body mass index.

**Table 2:** Results of comorbidities before COVID-19 and medication at the start of the study (post-COVID-19 condition) are presented as both a number and percentage within each group of participants. Statistics between groups was performed by using the chi-square 2-tailed test.

Parameters	Disorders before COVID-19		Taking Medication at the Start of Study (Post-COVID-19 Condition)	
	Telerehab (n = 67)	WL (n = 42)	Telerehab (n = 67)	WL (n = 42)
Diagnosis of any disease before COVID-19	24 (36%)	12 (29%)		
High blood pressure	2 (3%)	1 (5%)	16 (24%) Beta-blockers 7 (10%) other antihypertensive drugs	9 (21%) Beta-blockers 6 (14%) other antihypertensive drugs
Hypothyroidism	3 (5%)	1 (2%)	5 (8%)	2 (5%)
Asthma	5 (8%)	4 (10%)	17 (25%)	10 (24%)
Allergies Skin disease	3 (5%) 1 (1%)	1 (2%) 0	13 (19%)	5 (12%)
Psychiatric disorders: - Anxiety/depression - bipolar disorder - ADHD - PTSD	1 (1%) 7 (10%) 1 (1%) 2 (3%) 1 (1%)	0 2 (5%) 1 (2%) 1 (2%) 0	10 (15%) TCA/TTA 9 (13%) SSRI/SNRI 6 (9%) other psychiatric drugs	4 (10%) TCA/TTA 7 (17%) SSRI/SNRI 3 (7%) other psychiatric drugs
Inflammation/pain: - (poly)arthritis - chronic pain	1 (1%) 3 (5%)	1 (2%) 1 (2%)	4 (6%) anti-inflammatory drugs 5 (8%) NSAID 4 (6%) paracetamol 3 (5%) antiepileptics 4 (6%) opioids	2 (5%) anti-inflammatory drugs 1 (2%) NSAID 2 (5%) paracetamol
Gastrointestinal disease	3 (5%)	0	5 (8%)	7 (17%)
Kidney disease	0	1 (2%)		
Gynecological disease	1 (1%)	2 (5%)		
Vitamin deficiency	0	1 (2%)	5 (12%)	5 (8%)
Sleep disorders	0	2 (5%)	11 (16%)	8 (19%)
Others	3 (5%)	0	18 (27%)	8 (19%)

Abbreviations: Telerehab = Telerehabilitation; WL = waiting list; ADHD = attention-deficit/hyperactivity disorder; PTSD = post-traumatic stress disorder. TCA = tricyclic antidepressants; TTA = tetracyclic antidepressants. Other disorders include stress-related exhaustion syndrome (1 participant) and myalgic encephalomyelitis/chronic fatigue syndrome (2 participants). Other drugs include nasal sprays, anticontraceptive, antiviral drugs, and antithrombolytics.

**Table 3:** General linear model for ICF categories impaired in at least 75% of participants, which showed significant improvements within groups during the study. Time was chosen as a within-subjects factor and group as a between-subjects factors. Results are presented as the median, F, and *p*-value. Number of participants 109 at 8 weeks (intervention) and 81 at 6 months follow-up.

ICF Category	T1	T2	T3	Within-Subjects (Time)		Between-Subjects (Group)		Between-Subjects (Age)	
				F	*p* Value	F	*p* Value	F	*p* Value
Fatigability:									
Intervention	3	3		1.2	0.3	0.9	0.3	0.9	0.3
Follow-up 6 months	3	3	3	0.2	0.7	2.8	0.1	1.0	0.3
Energy and drive:									
Intervention	3	3		3.1	0.08	0.3	0.6	1.8	0.2
Follow-up 6 months	3	3	3	1.2	0.3	2.6	0.1	2.1	0.1
Concentration:									
Intervention	2	2		1.9	0.2	0.7	0.4	0.0	0.9
Follow-up 6 months	2	2	2	0.1	0.7	1.2	0.3	0.2	0.7
Short memory:									
Intervention	2	2		0.1	0.7	0.0	1.0	0.3	0.6
Follow-up 6 months	2	2	2	0.6	0.4	1.2	0.3	0.2	0.7
Muscle endurance:									
Intervention	2	2		4.3	0.04	1.1	0.3	0.7	0.4
Follow-up 6 months	2	2	2	0.0	1.0	0.3	0.6	0.1	0.7
Muscle strength:									
Intervention	2	2		3.1	0.08	2.9	0.09	0.4	0.5
Follow-up 6 months	2	2	2	0.05	0.8	1.1	0.3	0.1	0.8
Breathing functions:									
Intervention	2	1		3.6	0.06	2.6	0.1	0.4	0.5
Follow-up 6 months	2	1	1	1.4	0.2	0.8	0.4	2.4	0.1
Heart functions:									
Intervention	2	2		0.2	0.7	0.0	0.9	4.8	0.03
Follow-up 6 months	2	2	1	0.4	05	0.0	0.9	9.0	0.004
Pain in one site:									
Intervention	2	2		5.0	0.03	0.6	0.4	2.0	0.2
Follow-up 6 months	2	2	2	0.6	0.5	0.2	0.7	0.2	0.7
Pain in multiple sites:									
Intervention	2	1		9.1	0.003	0.9	0.3	1.3	0.3
Follow-up 6 months	2	1	1	0.4	0.5	0.4	0.5	0.9	0.3
Handling stress:									
Intervention	3	3		0.17	0.2	1.3	0.3	1.0	0.3
Follow-up 6 months	3	3	2	0.0	0.9	1.3	0.3	0.3	0.6
Remunerative employment:									
Intervention *	3	2		0.4	0.5	0.2	0.6	7.2	0.008
Follow-up 6 months **	3	2	2	0.0	0.9	1.3	0.3	1.1	0.3
Recreation and leisure:									
Intervention	3	2		2.6	0.1	0.0	0.9	2.9	0.09
Follow-up 6 months	3	2	2	0.6	0.4	0.1	0.7	0.1	0.8
Completing multiple tasks:									
Intervention	2	2		0.9	0.4	0.1	0.8	0.0	0.9
Follow-up 6 months	2	2	2	0.8	0.4	0.3	0.6	0.1	0.7
Climbing stairs:									
Intervention	2	2		5.2	0.02	0.0	0.8	2.6	0.1
Follow-up 6 months	2	2	1	0.3	0.6	0.3	0.6	2.3	0.1
Carrying objects:									
Intervention	2	1		0.5	0.5	0.0	0.9	1.3	0.3
Follow-up 6 months	2	1	1	0.1	0.8	0.0	1.0	0.6	0.4
Doing household:									
Intervention	2	1		0.6	0.4	1.0	0.3	5.3	0.02
Follow-up 6 months	2	1	1	1.4	0.2	0.4	0.5	3.1	0.08
Informal relations:									
Intervention	2	2		0.9	0.2	0.0	1.0	1.6	0.2
Follow-up 6 months	2	2	2	0.6	0.4	0.0	0.9	0.2	0.6

Abbreviations: T1 = median at the start; T2 = median after 8 weeks; T3 = median after 6 months; * N = 102 and 97 at T1 and T2, respectively, and ** N = 77, 73, and 76 at T1, T2, and T3, respectively.

**Table 4:** Parameters for breathing (mMRC and CCQ) are presented as mean, standard deviation, and range. Statistics within the group was performed by using a nonparametric Wilcoxon signed-rank test for data at the start and after 8 weeks, and a Friedman test for several dependent samples of data at the start, after 8 weeks, and at 6 months follow-up.

Questionnaires	At the Start		After 8 Weeks		Statistics after 8 Weeks		At 6 Months Follow-Up		Statistics for 6 Months	
	Telerehab (n = 67)	WL (n = 42)	Telerehab (n = 67)	WL (n = 42)	Telerehab (n = 60)	WL (n = 21)	Telerehab (n = 60)	WL (n = 21)	Telerehab (n = 60)	WL (n = 21)
mMRC	2 ± 1 0–4	2.3 ± 1.1 0–4	1.6 ± 1.1 0–4	2 ± 1.1 0–4	*p* = 0.001	*p* = 0.011	1.5 ± 1.0 0–4	1.4 ± 0.7 0–3	*p* < 0.001	*p* = 0.002
CCQ	20 ± 8.6 3–37	17.8 ± 9.5 1–37	15.1 ± 8.8 1–39	16.1 ± 8.5 1–29	*p* < 0.001	*p* = 0.124	15.6 ± 9.8 0–45	15.5 ± 8.6 1–31	*p* < 0.001	*p* = 0.016

Abbreviations: Telerehab = telerehabilitation; WL = waiting list.

## Data Availability

The data that support the findings of this study are available from the first author (IBL) upon reasonable request and after a completing approval by the Swedish Ethical Authorities (Etikprövningsmyndigheten).

## Supplementary Materials

The following supporting information can be downloaded at: https://www.mdpi.com/article/10.3390/jcm13040970/s1, S1: ICF codes presented in order according Figure 2, Figure 3 and Figure 4; S2: Presentation of content and time of team rehabilitation; S3: Detailed flow chart of the study cohort. No differences have been found between the groups except for age in the final cohorts (independent sample test). Abbreviations: TR = telerehabilitation group; WL = waiting list; BMI = Body Mass Index; S4: Participants’ evaluation of the treatment with the Credibility Expectance Questionnaire (CEQ) after 4 (median 4) and 8 (median 8) weeks of telerehabilitation, presented as median and minimum-maximum values, except for variable CEQ sum (mean and SD). * indicates significant differences within the group, a non-parametric Wilcoxon signed rank test, ** *p* < 0.01 and *** *p* < 0.001.
