# Supplementary material for: Improved Functioning and Activity According to the International Classification of Functioning and Disability after Multidisciplinary Telerehabilitation for Post-COVID-19 Condition—A Randomized Control Study
Source: J Clin Med. 2024 Feb 8;13(4):970. doi: 10.3390/jcm13040970 (PMC10889504; doi:10.3390/jcm13040970)
Supplement: Supplementary file 1 [file jcm-13-00970-s001.zip › jcm-2822671-supplementary.pdf]

Supplementary material 1 (S1). ICF codes presented in order according Figures 2-4.

Functions		Activities and participation	
b4552	fatigability	d240	handling stress
b130	energy and drive	d850	remunerative employment
b160	concentration	d920	recreation and leisure
b1440	short memory	d2201	completing multiple tasks
b1441	longterm memory	d870	economic self-sufficiency
b740	muscle endurance	d4551	climbing stairs
b730	muscle strength	d430	lifting and carrying objects
b735	muscles tone functions	d640	doing housework
b710	mobility of joint functions	d450	walking
b134	sleep functions	d4751	driving motorized vehicles
b440	breathing functions	d470	using transportation
b410	heart functions	d415	maintaining a body position
b152	emotional functions	d649	make the bed
b640	sexual functions	d750	informal social relationships
b535	gastrointestinal functions	d760	family relationships
b2801	pain body one site	d540	dressing
b2802	pain body multiple sites		
b2804	irradiating pain		
b2800	widespread pain		
b5500	body temperature		
b1562	olfactory functions		
b1563	gustatory functions		

Supplementary material 2 (S2). Presentation of content and time of team rehabilitation.

	Period 1	Period 2	Period 3
1. Psychoeducation:			
- Clinician: Introduction, presentation of data on the group level, lectures on the central nervous system´s regulation of autonomic functions, i.e., cardiovascular and pulmonary, including pathophysiology and treatment of orthostatic intolerance, regulation of sleep, pain pathways, autonomic impact on psychological aspects and restoring autonomic balance by body-mind therapy.	200 (8)	175 (8)	205 (7)
- Psychologist: Introduction to ACT, mindfulness, compassion, feeling of discomfort, background on brain regulation of emotions, theoretical background on yoga and relaxation therapies.	100 (4)	230 (10)	160 (6)
- Physiotherapist: Introduction and feedback about using the ExorLive application, BORG-scale, pelvis muscular functions for breathing regulation, cognitive-behavioural changes in rehabilitation.	110 (5)	25 (1)	70 (3)
- Occupational therapist: ergonomy, ExorLive Go application for ergonomic body positions, pacing.	135 (6)	125 (6)	90 (4)
2. Body-mind therapies:			
Breathing exercises	175 (10)	235 (12)	175 (9)
Mindfulness/ACT/compassion exercises	275 (14)	220 (14)	200 (10)
Relaxation	20 (1)	0	60 (3)
Body Awareness Therapy	45 (2)	50 (2)	40 (2)
Yoga and pelvic "root" lock	60 (4)	85 (4)	75 (4)
Qigong	215 (11)	170 (8)	140 (8)
Do-In	30 (2)	35 (3)	15 (1)
Physical exercises with or without pulse increase	120 (7)	170 (9)	60 (3)
Total time in minutes of rehabilitation interventions offered by the team during rehabilitation (according to the team´s dairy)	1605	1520	1290
Additional time*: 120 minutes. Introduction, presentation (the first day, 80 minutes) and summary of rehabilitation (the last day, 40 minutes)			
Total time in rehabilitation	1725	1640	1420

Period 1: 2021-05-10 – 2021-06-02, period 2: 2021-09-26 – 2021-10-28 and period 3: 2022-05-10 – 2022-06-30
Period 1 and 2: 48 hours per 8-weeks (3 sessions per week, 2 hours per session), breaks/reflexions 45-50 minutes per session. Approximately 1680 minutes of rehabilitation
Period 3: 44 hours per 8-weeks (3 sessions per week, 2 hours per session), breaks 45-50 minutes per session. Approximately 1540 minutes of rehabilitation.
* Every period had 120 minutes of introductions and presentations by participants on their own (the first day, 80 minutes) and summaries of the rehabilitation period (the last day, 40 minutes)
ACT= Acceptance and Commitment therapy.

Supplementary material 3 (S3). Detailed flow chart of the study cohort. No differences have been found between the groups except for age in the final cohorts (independent sample test). Abbreviations: TR= telerehabilitation group; WL= waiting list; BMI= Body Mass Index.

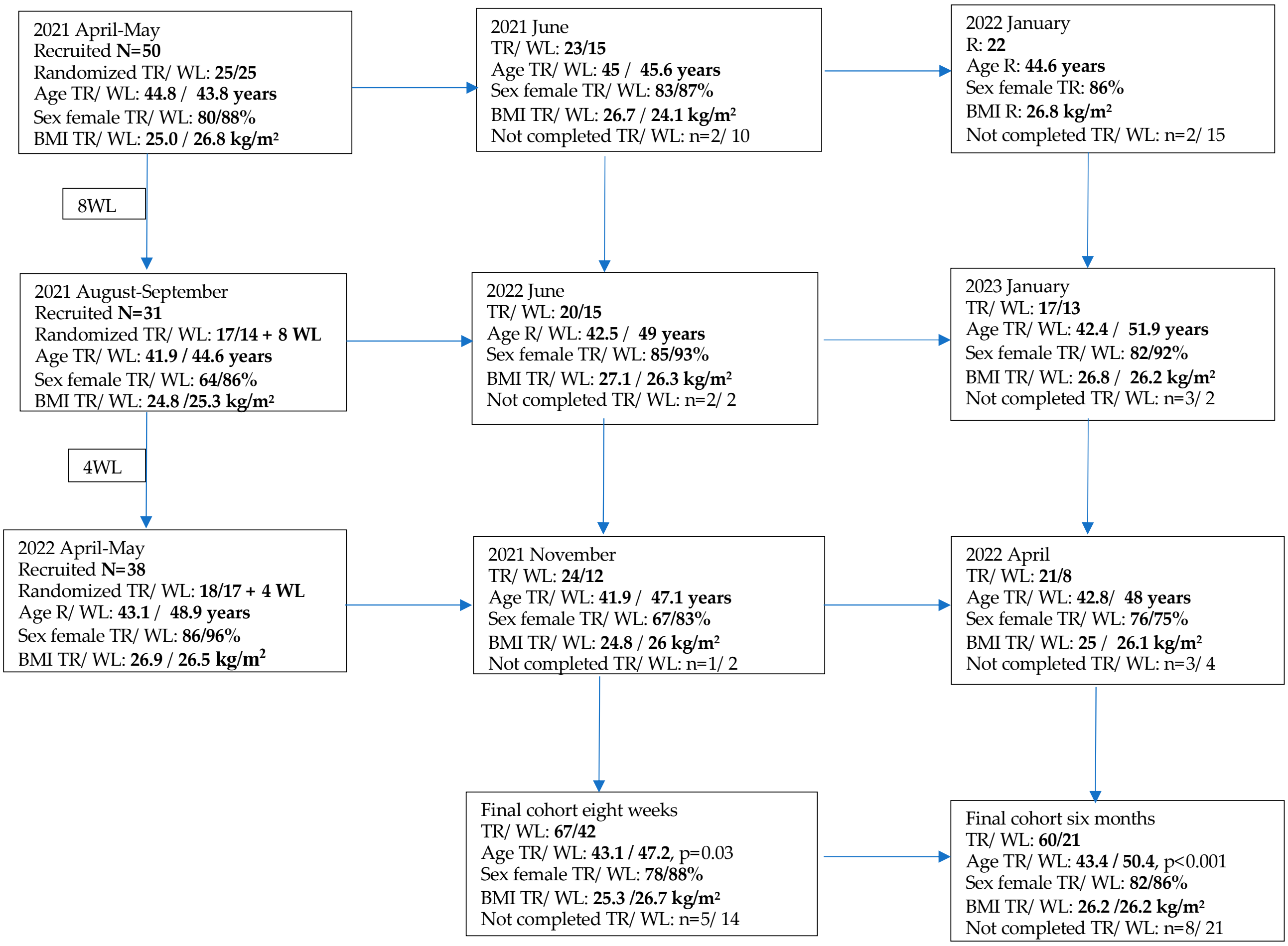

Supplementary material 4 (S4). Participants' evaluation of the treatment with the Credibility Expectance Questionnaire (CEQ) after 4 (median 4) and 8 (median 8) weeks of telerehabilitation, presented as median and minimum-maximum values, except for variable CEQ sum). * indicates significant differences within the group, a non-parametric Wilcoxon signed rank test, **p<0.01 and ***p<0.001.

Questions	After 4 weeks telerehabilitation	After 8 weeks telerehabilitation
1. How logical does the therapy offered to you seem? (Maximum 9)	8<br>3-9	8<br>5-9
2. How successfully do you think this treatment will be reducing in your symptoms? (Maximum 9)	7<br>3-9	7<br>3-9
3. How confident would you be in recommending this treatment to a friend? (Maximum 9)	9<br>2-9	9<br>3-9
4. How much improvement in your symptoms do you think will occur? (Maximum 100%)	50<br>0-100	50**<br>0-100
5. How much do you really feel that therapy will help to reduce your symptoms? (Maximum 9)	7<br>2-9	7<br>1-9
6. How much improvement in your symptoms do you really feel will occur? (Maximum 100%)	30<br>0-100	50***<br>0-100
CEQ sum (in mean and SD)	59 (26)	65 (26) **
